# Precision medicine and the problem of structural injustice

**DOI:** 10.1007/s11019-023-10158-8

**Published:** 2023-05-25

**Authors:** Sara Green, Barbara Prainsack, Maya Sabatello

**Affiliations:** 1 Section for History and Philosophy of Science, Department of Science Education, University of Copenhagen, Niels Bohr Building (NBB), Universitetsparken 5, 2100 Copenhagen Ø, Denmark; 2 Centre for Medical Science and Technology Studies, Department of Public Health, University of Copenhagen, Oester Farimagsgade 5, 1014 Copengagen, Denmark; 3 Department of Political Science, University of Vienna, Universitätsstraße 7, 1010 Vienna, Austria; 4 School of Social and Political Sciences, Faculty of Arts and Social Sciences, University of Sydney, Camperdown, NSW 2006 Australia; 5 Center for Precision Medicine and Genomics, Department of Medicine, Columbia University, New York, USA; 6 Division of Ethics, Department of Medical Humanities and Ethics, Columbia University, New York, USA

**Keywords:** Precision medicine, Personalized medicine, Structural Injustice, Equity, Healthcare model, Medical Matthew effects

## Abstract

Many countries currently invest in technologies and data infrastructures to foster precision medicine (PM), which is hoped to better tailor disease treatment and prevention to individual patients. But who can expect to benefit from PM? The answer depends not only on scientific developments but also on the willingness to address the problem of structural injustice. One important step is to confront the problem of underrepresentation of certain populations in PM cohorts via improved research inclusivity. Yet, we argue that the perspective needs to be broadened because the (in)equitable effects of PM are also strongly contingent on wider structural factors and prioritization of healthcare strategies and resources. When (and before) implementing PM, it is crucial to attend to how the organisation of healthcare systems influences who will benefit, as well as whether PM may present challenges for a solidaristic sharing of costs and risks. We discuss these issues through a comparative lens of healthcare models and PM-initiatives in the United States, Austria, and Denmark. The analysis draws attention to how PM hinges on—and simultaneously affects—access to healthcare services, public trust in data handling, and prioritization of healthcare resources. Finally, we provide suggestions for how to mitigate foreseeable negative effects.

## Introduction: the vision of precision in medicine

Although the genetic similarity among all human beings is 99.9%, precision medicine (PM) hinges on the idea that people’s bodies are different in such a way that “one size does not fit all” (Lovly and Carbone 2011). PM thus explicitly highlights the benefits of *treating patients differently*, presupposing that such differences can be objectively measured with new technologies. Yet, uncertainties remain regarding the scope of precision treatments because they are currently available only for a few patients with specific genetic conditions (Plutynski 2022). Ethical and social inquiries into the design and implementation of PM initiatives in several countries have underscored barriers to inclusion. Since most genetic studies and clinical tests have been conducted almost exclusively on White/European ancestry populations, the test accuracy and treatment efficacy are estimated to be lower for historically marginalized racial and ethnic populations (Geneviéve et al. 2020; Huey et al. 2019; Kurian 2010; 2021; Popejoy and Fullerton 2016).^1^ The problem is recognized in an editorial in *Nature* entitled “Precision medicine needs an equity agenda” (2021), which also describes initiatives to increase the diversity in genetic studies. However, while the equity agenda is often framed primarily as an issue of lacking *cohort diversity*, this paper aims to unpack wider *social and structural conditions* that are necessary for PM to benefit many and diverse patients.

Scholars in public health have long highlighted the importance of attending to structural causes to explain why population groups differ in disease prevalence and treatment outcomes (Bachur et al. 2018; Gerend and Pai 2008; Rose 1985). These include socioeconomic factors such as living conditions and barriers to healthcare access that are disproportionately present among historically marginalized subpopulations and affect health outcomes (Valles 2018). Similarly, scholars in law, bioethics, philosophy, and political science have stressed how structural biases systemically benefit some people while disempowering others (Marmot and Wilkinson 2005; Powers and Faden 2019; Schrecker and Bambra 2015; Young 1990). Yet, how structural factors relate to the equity effects of PM has not yet been the subject of much discussion. This paper starts to fill in this gap. We argue that robust realization of PM requires public trust in data handling, equitable access to healthcare services (not limited to the benefits of PM), and fair and efficient prioritization of healthcare services. With our focus on *structural injustice* in PM, we bring attention to how the structure of healthcare systems and societies can, in itself (and in addition to the inequity effects resulting from PM-specific research and implementation) result in asymmetrical effects of personalization in medicine. Moreover, we highlight aspects of PM that—even with good intentions to improve health care and outcomes—are likely to sustain, and exacerbate, existing health disparities. A commitment to inclusion and equity requires that structural inequalities are addressed not only in research in PM, but also by removing formal and informal barriers to the resulting healthcare applications, and by considering the best way to prioritize healthcare resources.

Our argument proceeds as follows: We first clarify the concept of PM ( "Personalized medicine and the turn to ‘precision’"), then analyze the structural and organizational implications of policies for the development and implementation of PM in three countries: the United States of America (henceforth U.S.), Austria, and Denmark (“Healthcare models and PM in the United States, Austria, and Denmark”). These countries have markedly different healthcare models and represent the combination of several characteristics that have bearing on the equitable realisation of PM: highly exclusive (the U.S.) and highly inclusive (Austria and Denmark) healthcare systems; highly digitized (Denmark and the U.S.) and less digitized (Austria) healthcare systems. Lessons from the three countries, from common concerns to challenges specific to the different contexts, underscore the importance of exploring the impact of structural issues on the equitable implementation of PM (“The organisation of healthcare as the foundation of PM”). We conclude with recommendations for strategies to minimize the risk of negative effects of personalization (“Discussion: Time to reconsider PM? and Concluding remarks”).

## Personalized medicine and the turn to ‘precision’

Precision medicine is the most current iteration of personalized medicine, a phenomenon that has been around for much longer. Important aspects of medicine have always been personalized, if we by this term understand the practice of considering the characteristics and circumstances of particular patients in diagnosis and treatment decisions. But what has changed are the types of evidence and information that are used for this purpose (Eyal et al. 2019; Prainsack 2017). In the aftermath of the Human Genome Project (1990–2003), the matching of drug treatments to genetic markers of patients became the paradigmatic case of personalized medicine (Juengst et al. 2016). Since then, the concept of personalisation further shifted to focus on a much wider range of practices and sources of information, with a stated goal of speeding up the translation of biomedical research to clinical practice (Green et al. 2022).

The more recent emphasis on *precision* can be interpreted as a terminological rebranding towards a more scientifically neutral name (Chan and Erikainen 2018). It emerged in the early 2010s as an expert group in the U.S. Institute of Medicine used the term Precision Medicine to refer to the multi-layered and data-driven understanding of medical personalisation (NAS 2011). This term was later also used in the launching of the $215 million Precision Medicine Initiative in the U.S. in 2015 (now called the *All of Us Research Program (AoURP)*, see NIH 2018). “Precision medicine” is therefore preferred in the U.S. context, while other countries (including Austria and Denmark) still predominantly use “personalized medicine” or even “stratified medicine”. Although the choice of terminology differs, the strategies subsumed under these labels are similar. Here, we thus use the PM abbreviation as a reference to these approaches.

PM is promised to provide patients with more targeted treatments and prevention strategies, and thus to reduce human suffering, health disparities, and healthcare costs (Cohn et al. 2017; Ministry of Health and Danish Regions 2016). Moreover, enhanced possibilities for individualized disease prevention are promised to result from genetic risk profiling, imaging technologies, and mass spectrometry that can make pre-disease stages visible before symptoms arise (e.g., Merlo et al. 2019). The different temporality of diagnostics, from a reactive to a *proactive* and *preventive* mode, is hoped to be particularly effective against so-called lifestyle diseases. For example, the Danish national strategy for PM underscores the benefit of genetic risk profiling by stressing that: “If you can give health advice early on to those at highest risk, it may be possible to better motivate lifestyle changes” (Danske Regione, 2015b, p. 10, our translation). This vision is also driving U.S.-based pilot projects, such as the *100P project* and *The Precision Medicine Screening Study* that collected unprecedented amounts of information on participants to provide individualized health recommendations (Perkins et al. 2018; Price et al. 2017). Similarly, in Austria, the nationwide and multidisciplinary Platform for Personalized Medicine was recently founded to “tailor prevention, diagnosis, and treatment of diseases based on molecular data and other relevant information obtained from patients”, so that “every patient can get the best possible prevention, diagnostic, and therapy at the ideal moment” (APPM 2022, our translation; see also Pot et al. 2020).

It is, however, important not to assume a smooth realisation of these visions: the effects of PM will depend on scientific developments, research inclusivity, and the willingness to address structural factors impacting who will benefit from the emerging knowledge and technologies. Since PM is still a technology and practice in development, our analysis will partly consist of the *anticipated effects*. This may raise the concern that the analysis is too speculative, as it is too early to tell what the impact of PM will be (Nordmann and Rip 2009). Yet, an “ethical assessment” of emerging technologies may be particularly useful if it is conducted early enough to have an impact on the development or implementation of new healthcare strategies. Moreover, such an assessment can be both imaginative and empirically grounded, e.g., on an analysis of the effects of initial developments or on historical and situated knowledge about the contexts of implementation (Lucivero et al. 2011). We take a step in this direction by scrutinizing how the effects of PM depend on the structure of healthcare systems, exemplified through a comparison of the healthcare models and PM initiatives in the U.S., Austria, and Denmark (see Table 1). These three settings represent different case comparisons in their approach to healthcare (universal and publicly funded (Austria and Denmark) v. private healthcare systems (U.S.)) and coordinated investments into PM (public and private in U.S. and Denmark v. absence of such a coordinated programme in Austria). We highlight key issues of concern that are important to discuss at an early stage of—or preferably before—implementation, while the opportunities to avoid or mitigate foreseeable negative implications of PM still exist.

**Table 1:** Factors that influence the development and effects of PM in the U.S., Austria, and Denmark

	United States	Austria	Denmark
Basic features of healthcare model	* Privatized, for-profit with limited public programs (e.g., Medicare, Medicaid) * Highly fragmented, inefficient at macro-level, costly & unaffordable * High-tech medicine & digitized * No insurance requirement * Access tied with healthcare plan * Medical bills and bankruptcy are common	* Bismarck-style system available to everyone, alongside voluntary private health insurance * Multi-level, public-led agencies involved * Limited high-tech & digitalization, basic electronic health records system * Compulsory insurance * Out-of-pocket payments are limited and few	* Beveridge healthcare system: health care provision is funded directly by income tax * High-tech & digitized (centralized) * General practitioners as gatekeepers to specialized care * Optional private insurance to cover co-payments and additional services * Out-of-pocket payments are limited and few
Coverage & expenditure	* Approximately 20% of GDP * Coverage is insurance-dependent and limited * Treatment-focused, not preventative * Expanses and affordability tied with employment-based insurance (if available) and states’ support	* Approximately10% of GDP * Universal coverage * Insurance contributions are proportionate to income * Wide range of diagnostic, preventative, and therapeutic options available in the basic statutory insurance basket	* Approximately 10% of GDP * Universal coverage * Tax contributions are proportionate to income * Basket of services predetermined; coverage of based on cost-effectiveness evaluation
Initiatives of Precision Medicine	* National programs, The *All of Us Research Program* and *Cancer Moonshot*, funded by the National Institutes of Health (NIH) * Public–private collaborations through public funding * Local/university-based precision medicine programs	* No national program * The Austrian Platform for Personalized Medicine, funded by the Austrian’s Federal Minister of Education, Science and Research * Initiatives to align research and funding activities in PM in Europe, e.g., ICPerMed	* National strategy for personalized medicine * Population-wide initiatives, including the National Genome Center, that extend existing digitized registers as research resources * Collaboratory structures, e.g., the Trial Nation, for global investment
Barriers to PM development and/or utilization	* Underserved/minoritized populations underrepresented in research cohorts and in implementation rates of genomic applications (particularly Black/African American, Hispanic, and Indigenous populations) * Limited access for uninsured and underinsured patients * Fewer genomic centers in rural settings	* Low degree of digitization of health care infrastructures * Comparatively low public trust in data handling compromise options for collection and integration of genomic and other health data	* Limitations in public healthcare resources, workforce and specialized genetic knowledge among practitioners

## Healthcare models and PM in the United States, Austria, and Denmark

To unpack the importance of structural factors for the implementation and effects of PM, we examine the characteristics of PM initiatives and healthcare structures in the U.S., Austria, and Denmark in the following. Table 1 gives an overview of key aspects that influence inclusion and exclusion into PM.

### PM in the United States

The U.S.’s investment in high-tech and genomic medicine is high compared to other countries (Anderson et al. 2019). Since the late 1980s, and especially since the completion of the Human Genome Project in 2003, there have been growing efforts to improve health outcomes via individually-tailored approaches (Juengst et al. 2016). The 2015 announcement of President Obama to establish a national precision medicine initiative (the *AoURP*) has galvanized the rhetorical transition and cemented the financial and conceptual investment in PM as a new model for healthcare. The *AoURP* was followed by *The 2016 Cancer Moonshot*, another nationally-funded initiative to promote precision oncology (21st Century Cures Act, 2016; National Institute of Cancer 2016), and an increasing number of healthcare facilities are conducting precision medicine within their organizations, and across the landscape of medicine—including also neurology, prenatal screening, cardiovascular disease, nephrology, and psychiatry (Bresnick 2016; Nestor et al. 2018; Stein and Smoller 2018).

AoURP directly engages the general public (and not only patients seeking treatment) in the production and sharing of health information, including genetic data and electronic health records. *AoURP* is thereby intended to speed up the digitalization and integration of health data resources (Vegter et al. 2022) while emphasizing research inclusion and rectifying the country’s broken healthcare system (AoURP investigators 2019; Hoffer 2019; Sabatello and Appelbaum 2017). These are important national endeavors given that, unlike other high-income countries, the U.S. healthcare model does not offer a universal, national healthcare system (some exceptions exist, e.g., Medicare for the elderly; see Table 1 for key characteristics in the U.S., Austria, and Denmark). Moreover, although the system is invested in high-tech medicine and highly digitized,^2^ it is notorious for being fragmented, costly, and inefficient at the macro-level (Ballou and Landreneau 2010; Barthold et al. 2014; Fuchs 2018). It is largely controlled, regulated, and administered by private for-profit health insurance and pharmaceutical companies. Despite being one of the largest industries in the country, accounting for almost 20% of the GDP (CDC, 2018), it is inequitable, unaffordable, and inaccessible for significant segments of the population. It has also been oriented primarily toward male and White “standard patients” (Tutton 2014). Studies indicate that Black/African American, Hispanic, and Indigenous populations are more likely to be poor, score lower in their social determinants of health compared to Whites, and are less likely to be insured (Sohn 2017). Despite the 2010 adoption of the Patient Protection and Affordable Care Act (the ACA) which address some of the inequalities, medical bills remain a common cause of financial hardship and bankruptcies (Himmelstein et al. 2019), and they occur at disproportionately high rates among already marginalized racial and ethnic communities (Ehlers and Hinkson 2017).

There are reasons to expect that the privatized healthcare model in the U.S., including its historical biases and structural racism, will affect access to and utilization of PM. Studies indicate that clinicians are less likely to recommend genetic testing to racial/ethnic minorities and that system-level barriers may affect their decision-making about genetic testing (McCarthy et al. 2016). These include: lack of access to genetic counselors, inconvenient access to genetics centers, perceived costs to both patients and providers (especially in the absence of health insurance coverage), and unreimbursed time spent on ordering genetic tests (Mikat-Stevens et al. 2015). Such barriers are more likely to occur in rural and low-income locations, which are disproportionately inhabited by historically marginalized racial and ethnic communities (NASEM 2018; Creamer 2020). The underrepresentation of marginalized racial and ethnic communities in genomic research further affects the generalization of knowledge of PM, including the accuracy of genetic testing and pharmacogenomics (Kurian et al. 2021; Popejoy and Fullerton 2016). Although there are ongoing efforts to enroll diverse populations into PM research programs (e.g., AoURP investigators 2019), translational efforts are likely to be slow, and a recent review documents lower implementation rates for genomic medicine among underserved populations (Khoury et al. 2022, see also “Equity in access”).

### PM in Austria

Unlike the U.S. and Denmark, Austria has no coordinated programme of public and private funding to promote PM. However, it has initiatives to facilitate multidisciplinary collaboration in research to support funding applications in this field. In the mid-2010s, the Austrian Federal Ministry of Education, Science, and Research funded the establishment of the Austrian Platform for Personalized Medicine, a platform for collaboration and coordination among the three medical universities in the country and a research center dedicated to molecular medicine. It brings together physicians, medical and social scientists, science communication experts, patient advocates, and representatives of the pharmaceutical industry in Austria and beyond. In 2020, the platform had 140 individual and 16 institutional members, including non-profit organisations such as universities, extramural research institutions, patient advocacy groups, scientific associations, and the association of the Austrian pharmaceutical industry (Pot et al. 2020). In addition, Austrian research funders, such as the Austrian Science Fund (FWF) and the Vienna Science and Technology Fund (WWTF) participate in, or run, funding calls focused on PM, including the ICPerMed programme (www.icpermed.eu). ICPerMed was established to align research and funding activities in the area of PM in Europe and ultimately also at the international level.

Also unlike in the U.S. and Denmark (see Table 1), a major obstacle for PM in Austria is public concern regarding efforts to digitize and harmonize health data infrastructures. Throughout the 2010s, the rollout of a nationwide system of electronic health records (*elektronische Gesundheitsakte, ELGA*) was initiated to systematically link patient information. However, the purpose of the data captured within the ELGA system is still mainly clinical (e.g., drug prescription, lab results, medical images) and only accessible to health service providers. Patient-specific genomic information is generally stored at the diagnosing institution without transfer to centralized databases. Despite this very limited integration of digitized health information, and options for individual patients to opt out, the introduction of the ELGA system had still evoked much public concern, as well as resistance from doctors and other healthcare professionals (Hackl et al. 2011; Hofmarcher 2008). This is partly due to a traditionally high level of concern around data privacy in German-speaking countries, which finds expression also in the Germanic notion of informational self-determination (Voigt et al. 2020; see also Prainsack and Gmeiner 2008).

In Austria, the lack of public trust in data handling is not grounded in the issues of inequality in access and discrimination, as is the case for the U.S. Overall, the Austrian healthcare system is highly inclusive, and Austria is considered to have one of the best and most inclusive healthcare systems in the world (Bacchus and Moir 2019). As a Bismarck-style system, its core funding mechanism is mandatory social security contributions that are directly or indirectly related to employment via a system of compulsory insurance. Some services are funded through taxation and co-payments such as for hospital stays, prescriptions, etc. that are progressively related to income while ensuring universal healthcare coverage for all (Austrian Federal Ministry of Labour, Social Affairs, Health and Consumer Protection 2019). People’s contributions to health insurance are determined by their income, and access to services is strictly based on medical needs and not the ability to pay.^3^ This is in line with the solidaristic spirit characteristic of continental European healthcare systems, also including Denmark which we discuss next.

Although a very generous basket of services is covered by mandatory insurance, there is a growing sector of private health insurance for those who want additional services. The Austrian healthcare system, unlike Denmark, has no strict gatekeeping system: in many situations, people can access doctors, often also specialists, without obligatory referrals by their primary family doctor (neither are they bound to have only one family doctor). ‘Shopping around’ for second, third, or fourth opinions is not rare. It is also for this reason that Austria’s healthcare system, while overall lauded as very generous and high-quality, is seen as simultaneously suffering from the problem of under- and overtreatment. Given this structural background, a concern is that PM may further stimulate the private insurance market and doctor-shopping (we discuss this issue further in “Prioritization of healthcare resources”.

### PM in Denmark

Denmark has been described as “an epidemiologist’s dream” (Frank 2000) and as an ideal context for realizing PM (Hoeyer 2019; Hillersdal and Svendsen 2022). The small welfare state is considered one of the most digitized countries in the world (United Nations 2020), and integrated health databases provide individual-level and lifelong linkage to all records via a personal identifier, called a CPR number. Denmark is in the lead worldwide in the use of electronic health records in primary and secondary care (Schmidt et al. 2019). A national strategy for PM was published in 2015 and updated in 2016 and 2021, describing the ambition to implement PM in virtually all aspects of health care, from oncology to prevention of common diseases (Danske Regioner 2015a; 2015b; Ministry of Health and Danish Regions 2016; Sundhedsministeriet og Danske Regioner 2021).

To realize this vision, additional population-wide initiatives for collection and integration of genomic and health data are considered necessary. A flagship is the National Genome Center, established in 2019 as a new agency within the Danish Ministry of Health, with financial support from the foundation of the largest Danish pharmaceutical company (Novo Nordisk Foundation 2018; Danish National Genome Center 2019). The Danish National Center is planned to store genomic data on the whole population, starting with whole genome sequencing of 60,000 patients from 12 selected patient groups, to be used for both clinical and research purposes. One of these groups is cancer patients and multi-site experimental trials in precision oncology are established, often sponsored by pharmaceutical companies. An important initiative to facilitate such public–private partnerships was the establishment of *Trial Nation* in 2018, a merger of previous initiatives to attract global investments in clinical trials in Denmark by providing a single entry to comprehensive health data.^4^ The initiative received further support with a new Life Science Strategy, published by the Danish government (Regeringen 2021).

The basis for the uniqueness of the Danish health databases is a very high degree of public trust in the government for data handling (see “Public trust in data handling”), as well as a general commitment to the welfare state’s core principles of solidarity and equality. Like Austria, Denmark has universal health care coverage. But the Danish system is structured as a single-payer national health service model (or Beveridge model), where the majority of health care expenses (about 84%) is covered by taxes that are progressively adjusted to income. The remaining 16% are financed via patient co-payments for some services, including some prescription medicine, dental care, and physiotherapy (Healthcare Denmark and Ministry of Health 2017). Co-payments can in some circumstances be covered by public subsidies or via supplemental employer-paid or private health insurance.

The hospital sector is largely public and financially supported through block grants and reimbursement schemes from the government. 99% of Danish residents are registered with a specific General Practitioner (GP), who (unlike the Austrian system) serves as gatekeepers to specialized care (private and public), except for the emergency department and dental care (Schmidt et al. 2019). GPs are private entrepreneurs, but they work under a government-based contract and receive payment through a centralized remuneration system. National legislation ensures a set of common patient rights, including free choice of hospitals and GP, and maximum waiting time guarantee on diagnosis and treatment. If specialized treatments for life-threatening diseases are not provided by a Danish hospital, patients can be referred to hospitals abroad, conditioned upon approval by the Danish Health Authority.

To optimize and streamline use and access across hospitals and regions, new treatments are evaluated for safety and clinical effect (measured as quality-adjusted life-years) by the Danish Medicines Council; they are only approved when documented effects are deemed to be “reasonably proportionate” to the costs (Medicinrådet 2021, p. 4). The regulation of approval based on evaluation of cost-effectiveness was introduced to ensure that public health care resources are effectively spent. Against this background of centralized prioritization efforts, the high cost of many PM treatments has made such issues of prioritization increasingly visible also to the public in Denmark (see “Prioritization of healthcare resources”).

## The organisation of healthcare as the foundation of PM

The interconnection between PM implementation and the structure of healthcare systems and societies can be highlighted by exploring similarities and differences in the respective countries. Below, we argue that attention to healthcare structures with respect to the following domains is critical for robust implementation of PM: (i) Public trust in data handling, (ii) Equity in access, and (iii) Prioritization of healthcare resources. The main points are summarized in Table 2 and clarified in the following sections.

**Table 2:** Concerns associated with PM in the U.S., Austria, and Denmark

	United States	Austria	Denmark
Concerns associated with PM	* Non-transferability of PM findings across racial and ethnic groups due to underrepresentation of underserved racial and ethnic individuals in PM cohorts	* Slow development of data infrastructures for PM due to concerns about data privacy and surveillance	* Destabilization of the public trust in the welfare state due to increasing commodification of health data for commercial purposes
	* Exacerbating existing inequities among underserved and minoritized populations due to structural barriers (e.g., insurance-dependent access, racism)	* Stimulation of the private insurance market/doctor-shopping	* Increasing problems with medical Matthew effects, as consumer demands for genetic services put pressure on healthcare resources
	* Rising costs of health insurance * Risk of misuse and reification of racial categories through emphasis on genetic differences between groups		
	High costs of targeted treatments		
	Uncertainty about cost-efficiency of some applications		
	Neglect of structural factors that contribute greatly to inequality in health		

### Public trust in data handling

The realization of PM depends on collection and integration of large amounts of genomic and health data. Thus, public trust in organisations to securely handle the data—while protecting privacy, confidentiality, and equal opportunities—is a topic that exposes the importance of the national context for handling health data (Lee 2021; Wadmann et al. 2022). As discussed in “Healthcare models and PM in the United States, Austria, and Denmark”, the degree of digitization of the Austrian healthcare system is relatively low. Public and stakeholder resistance has accompanied, and stymied, attempts to create digital infrastructures—even if they combine only a very limited range of information. The absence of a national strategy for PM in Austria can be partially explained by significant public concerns about undue surveillance and possible violations of privacy rights associated with the centralised digital storage and widespread use of genetic and genomic information (Prainsack and Gmeiner 2008; Schumann et al. 2021). A lack of trust in institutions that handle genomic and health data can thus hinder the development of PM.

In the US, large consortiums of genomic and PM research are funded by the National Institute of Health (NIH). Yet, public funding is also explicitly geared to foster collaborations and data use by private, for-profit, and pharmaceutical companies. Specifically, NIH policy requires data sharing of samples collected through NIH-funded studies; the *AoURP* explicitly offers a platform for private-sector PM researchers (and others) to access information once certain screening requirements are met. Although such public–private partnerships are viewed as instrumental for scientific developments, this may also impact program enrollment. Studies of the general public, including in particular historically marginalized communities, have found distrust particularly in private, for-profit organizations (Kaufman et al. 2016; Sabatello et al. 2019; see also Milne et al. 2021; Wellcome Trust 2016). Whether and how such views affect actual enrollment, data collection, and retention is unknown, though there are reasons to believe it may, especially among underrepresented populations. Studies of African American individuals, for example, show very low levels of trust in the privatized healthcare system and its inequitable provision of access to health services, including the opportunity to access scientific benefits (Musa et al. 2009; Passmore et al. 2019). A study of public attitudes to research participation in the U.S. also showed that experiences of limitations to access to healthcare influence how patients perceive the benefits of research participation (Kraft et al. 2018). Thus, systemic changes that consider and address the causes of distrust, including incidents of mistreatment in research and continuous bias in all aspects of life are essential for creating healthcare systems and PM programs that are worthy of participants’ trust (Claw et al. 2018; Passmore et al. 2019; Sabatello et al. 2020; Ulrich et al. 2013).

Denmark is generally characterized as a country with a high degree of public trust in the government, and health data can generally be reused for clinical purposes and research without informed consent (Hoeyer 2018). Health data are often conceptualized as a currency that strengthens the reciprocal relation of exchange of goods between citizens and the welfare state: citizens donate data and in return benefit from research and healthcare services (Jensen and Svendsen 2021; Terkildsen et al. 2020). The potential of integrating such information with existing health databases has been highlighted as a “goldmine” for the welfare state, and as something that makes Denmark ideal for realizing PM (Hoeyer 2019; Regeringen 2021). Yet, the trust is not unconditional. PM comes with increasing concerns about who can access Danish health data, particularly genomic data (Svendsen 2019). As part of the establishment of the National Genome Center, a proposal was made for an amendment to the Danish Health Law, stating that genome sequences, like other forms of health data, could be conducted as part of treatment and stored in the center without written consent. The suggestion stirred public debates and strong objections (O Cathaoir 2019). A modified amendment was adopted in July 2018, which makes written consent to genomic analysis and biobanking mandatory. This debate demonstrates the high sensitivity awarded to patient genomic data (different from other patient information) and thus not straightforwardly as a common resource (Svendsen 2019; Svendsen and Navne 2022). Yet, while the legislative change to include an opt-out option for genetic data ensures a higher degree of autonomy for individual patients, it fails to address public concerns about profit motives that primarily benefit industry stakeholders (Skovgaard and Hoeyer 2022). Thus, while the implementation of PM is accelerated through trust and collectivity in the Danish welfare state, PM–like in the U.S.–comes with increased repurposing of patient data through public–private partnerships involving commercial interests, which may destabilize trust relations. Snell, Tarkkala, and Tupasela frame this as a “solidarity paradox”: PM is made possible through the patients’ and public’s solidaristic sharing of data, but the underlying data economy contradicts those very same values (2021). These issues are given additional attention in the updated Danish strategy for PM, where the benefits to patients are further emphasized (Sundhedsministeriet and Danske Regioner 2021). Yet, in the same year, the government issued a “Strategy for Life Science” emphasizing the benefits of ensuring “fast and easy access” to Danish health data for industry partners (Regeringen 2021). This underscores the importance of critically discussing what are legitimate uses of health data, as PM needs to be facilitated in a way that does not undermine the founding trust in institutions to handle genomic and health data.

### Equity in access

PM initiatives often emphasize the importance of cohort diversity, not least as a step to rectify existing biases. The American *AoURP*, e.g., has successfully promoted racial and ethnic diversity since its inception and achieved a highly diverse enrollment of populations that have been historically underrepresented in biomedical research (AoURP investigators et al. 2019). However, the issue of structural injustice is not only a question about research inclusivity. Whether and how investments in PM will translate into equal health benefits for all depends on the extent to which increased precision of diagnostic tools will result in better access to treatment and preventive health care for marginalized groups.

As emphasized in “Healthcare models and PM in the United States, Austria, and Denmark” (see also Table 1), issues of access, costs, and bias are likely to be paramount in the U.S. healthcare system. PM is a highly specialized area of medical research and practice, and there is considerable variability and lack of transparency among private and public health insurance programs with regard to which genetic services will be covered, how they define “clinical utility” to cover PM services, and what the out-of-pocket costs for privately insured individuals will be (Council on Medical Service and the Council on Science and Public Health 2017). With Black/African American, Hispanic, and Indigenous populations more likely to have public health insurance and limited resources (Creamer 2020; Sohn 2017), access for these communities to PM is particularly limited. Additionally, although the environmental aspect of PM is key for tailored interventions, the U.S. healthcare system post-ACA has remained focused on diagnosis and treatment rather than prevention (Manchikanti et al. 2017). With no comparable investment in public health services and improvement of social determinants of health, the benefits of PM are unlikely to equally accrue to members of society or improve population health outcomes (Bayer and Galea 2015). A recent review of genomic implementational studies confirms this concern by showing that implementation rates for genetic applications to treat hereditary breast and ovarian cancer, familial hypercholesterolemia, and Lynch syndrome, are significantly lower for marginalised ethnic communities, as well as for disadvantaged groups with lower income, education, and who are un- or underinsured (Khoury et al. 2022).

As the Covid-19 pandemic has brought attention to the robustness of healthcare systems, people’s ability to trust that they will receive good quality healthcare independent of their ability to pay would likely be a factor linked to people's willingness to share data and to participate in research. Moreover—and this speaks to an even deeper structural factor—knowing that one’s basic needs, such as housing, education, or transportation will be satisfied by public service provision also lowers the risk to suffer serious personal or financial harms emerging from participating in medical research (Yu et al. 2013).

The structure of the healthcare systems in Austria and Denmark exemplifies how this problem can be mitigated by making access to healthcare relatively independent of financial status and insurance coverage. In Austria and Denmark, individuals with a medical need for an approved precision treatment would be expected to be equally covered, regardless of financial status. In these contexts, the solidaristic sharing of risks and costs via the organisation of the healthcare system means that it is less likely than in privatized healthcare systems (as in the U.S.), that only the already well-off will benefit from PM. Thus, the possibility of the PM benefiting all strongly depends on whether wider health care and societal structures are driven by profit considerations or principles of solidarity. However, as we emphasize below, the expenditure for developing PM, including the pricing of targeted treatments and public funding for data infrastructures and research investments, makes it important to address issues of prioritization of healthcare resources for optimal public benefit. If issues of prioritization are not considered, PM may result in downstream structural injustices even in healthcare systems that currently have universal healthcare coverage.

### Prioritization of healthcare resources

PM is often assumed to be more cost-effective than “traditional” medicine because treatments will be more efficient and disease prevention will be improved. Yet, cost-effectiveness is often assumed in political reports, rather than documented through examples. This holds in particular for individualized risk profiling, as discussed below. Moreover, improving cost-efficiency not only hinges on the scientific achievement of developing “more precise” strategies for treatment and prevention. It also depends on ensuring that mechanisms for the pricing and marketing of drugs do not undermine existing healthcare systems. If costs increase significantly, without corresponding boosts in efficacy, public healthcare systems may become under pressure, thus stimulating a market for additional health insurance that not all citizens can pay for.

PM is often presented as the straightforward choice of selecting the best treatment for specific patients. But the evaluation of the relative value, relevance, and cost of PM treatments for different patient groups can be highly challenging. Despite the hype, the efficacy of many targeted treatments is still uncertain, even for the frontier domain of precision oncology. The benefits of many targeted cancer treatments currently amount to a few additional months of “progression-free survival” for a small subset of cancer types (Plutynski 2022). Moreover, targeted treatments are currently available only to a highly selected group of “precision patients”, such as those who fit the requirements of industry-sponsored clinical trials by having a specific mutation and a disease progression status acceptable to the trial protocol (Dam et al. 2022; Hillersdal and Svendsen 2022). For countries with universal healthcare coverage, “precision patients” selected for targeted cancer treatments need not be socio-economically privileged. In such situations, precision treatment is in principle compatible with solidarity–a concept that applies even though targeted treatments currently do not exist for all cancer types and thus would not benefit all patients. Yet, the high cost of PM treatments and stratification of existing patient groups via genetic testing make prioritization issues both more challenging and more visible to the public. This could fuel demands for increasing services that can drain public funds and stimulate a market for additional private health insurance.

In Denmark, for example, the Medicines Council recently approved a new ovarian cancer treatment only for patients with BRCA mutations, despite expectations that the new treatment might be more efficient for all ovarian cancer patients, compared to standard treatment. The basis for Council’s decision was that for patients without BRCA mutations, the added value was found to not reasonably correspond to the high additional cost of treatment. This decision was experienced as unjust because more effective PM treatments may only be available to a subset of patients who, at least at the phenotypic level, have the same disease. While the practice of inclusion and exclusion in this context is not tied to socio-economic disparities, we highlight the example to illustrate how structural divisions may result as downstream effects of increasing public expectations to PM and drug markets capitalizing on these. Although other, non-structural inequities clearly exist, these structural aspects have not received sufficient attention in connection with PM. In Denmark, the Medicines Council’s decision spurred public debate, where frustrated patients stressed the availability of the drug in other countries and compared the prioritization in Denmark to a lottery (Müller and Pam 2019a; 2019b). This reveals the role of the global market in influencing patients’ expectations of public healthcare (see also Hillersdal and Svendsen 2022), and how patient activists and citizens may react by calling for additional healthcare coverage within or beyond the public healthcare system.

Several treatments are approved in other countries but are considered too expensive to be covered in the Danish tax-financed healthcare system. Strikingly, however, attention is often turned to the lack of coverage, rather than to the basis for the high drug prices. The American media company CNN recently reported on the tragic case of a Danish toddler with an aggressive form of spinal muscular atrophy (SMA), who does not have access to a new pharmacogenetic treatment, Zolgensma, that could potentially save her life (Karimi 2021). Zolgensma is the world’s most expensive drug, currently estimated to cost 1.9 US$ for a single treatment (Nuijten 2022). While approved in the U.S. (pending the existence of and approval by health insurance), the Danish Medicines Council found the costs too high, compared to the clinically documented benefits. Similar considerations made the Council recommend another similar treatment, Spinraza, but only to patient groups with genetic variations that increase the expected benefits from treatment. This led to protests from clinicians and patient groups, as the new strategies of stratification were based on considerations of cost and not only medical benefits (Wadmann and Hauge 2021). In Austria, in contrast, the costs for treatments with Zolengsma are covered, with no co-payments for patients. Because of the significant costs of the treatment, the costs are borne by the Federal Health Agency (*Bundesgesundheitsagentur*), an organisation under the authority of the Federal Ministry of Health whose main role is to distribute federal funds for the financing of public hospitals. While there has been no wide public debate about the coverage of excessively expensive treatments, there is one about the injustices stemming from the fact that the same doctors and healthcare facilities are treating ‘regular’ patients and those with private top-up health insurance, and that they prioritize resources with the latter. The recent shortage of doctors and other healthcare professionals, together with increasing prices for targeted PM treatments, are exacerbating this trend. Unless the high costs of PM treatments are outpaced by effectiveness, economic and public pressure on universal healthcare systems will increase. The added pressure on public funds could stimulate markets for additional, private health insurance. In this case, downstream effects may be increased structural injustices even in healthcare systems that currently uphold solidarity and value equality in access.

The examples above highlight the importance of discussing how and whether PM could become standard care when additional costs do not straightforwardly translate into additional benefits, and how one can avoid some patients feeling left behind. This can happen when hyped precision treatments can be made available only to some patients, or when structural inequalities are stimulated as a downstream result of the increasing cost of targeted treatments. Zolgensma is the most striking example. The price of the drug is not based on the cost of development of the treatment, but on the company’s estimation of the long-term healthcare costs for children with spinal muscular atrophy in the U.S. (Karimi 2021). This pricing strategy stands in stark contrast to the widespread political expectation that PM will reduce healthcare costs. Meanwhile, the company behind Zolgensma (Novartis) has launched a lottery-style program, where a number of free doses are offered to countries where the drug is not currently approved. This may seem like a generous offer to help some patients with severe SMA (Karimi 2021), but this “compassionate use” strategy in effect puts pressure on countries with universal coverage to approve overpriced treatments (Moe 2020). As mentioned, the current marketing of expensive targeted treatments may also create a privatized global market for PM, where resourceful citizens will increasingly buy additional private health insurance to get access to more advanced therapies. If unaddressed, this problem will likely increase as more genetically targeted treatments become available.

A much stricter regulation of drug pricing, as well as of pharmaceutical profit making more broadly, are not topics prominently discussed in connection with PM. On the contrary, the received view is that pharmaceutical companies need to be given incentives to keep investing in drug development as molecular testing is reducing the market size for many drugs (for an overview see Triveedhi 2018). We are thus left with the question of how such treatments should be funded in future healthcare systems. In a recent paper on solidarity arguments in medical contexts, van Till and colleagues argue that although normative support for public funding of expensive treatments is often justified by reference to solidarity (addressing patients in need), “it does not necessarily require such funding when it conflicts with requirements of justice or when it may undermine a public healthcare system. And while allowing individual patients to pay for treatments that are not (yet) reimbursed may help alleviate their needs, it introduces inequality and may deteriorate solidarity between patients” (van Till et al. 2022, p. 8). As resources are limited, the sustainability of public healthcare systems depends on constraints on funding of treatments that are insufficiently cost-effective.

A response to this criticism might be that PM overall will lead to reduced suffering and healthcare costs, because it will enable more efficient *disease prediction* and *prevention*. Such visions are highlighted in political strategies, where it is envisioned that action can be taken before an unhealthy lifestyle results in symptomatic disease with associated healthcare costs (e.g., Danske Regioner 2015b). Indeed recruitment of participants for data donation is often conducted through the promotion of the opportunity to access health data, learn about disease risk, and take action to stay healthy (National Institutes of Health 2015; consent form of All of Us 2018; Price et al. 2017). However, there is to date no compelling evidence that individualized prevention strategies are more effective than ‘traditional’ prevention of common diseases. Studies so far fail to demonstrate that individualized risk profiling significantly motivates lifestyle changes or impacts health outcomes (Hollands et al. 2016; Vogt et al. 2019).

Moreover, as also emphasized by pioneers of PM, successful individualised disease prevention requires longitudinal monitoring, follow-up testing, and health counseling (Price et al. 2017). Even if prices of molecular testing will reduce, individualized counseling and continuous health monitoring require extra resources that may not be met by comparable benefits. Strikingly, the scientific wellness startup Arivale, co-founded by Leroy Hood in connection to the P100 pioneer project, closed due to: “the simple fact that the cost of providing the service exceeds what our customers can pay for it” (Bishop and Soper 2019). Such services often go beyond what standard health insurances are willing to pay. As a result, the benefits may be reserved only for a few resourceful customers, who are statistically at lower risk than the general population.

Universal access to risk information and health counseling alone would not address all challenges either. Because preventive PM is being promoted as a high-tech, proactive, information-intensive, and participatory direction of medical care, attention should be paid to differences in health literacy and digital capabilities among citizens (Alami et al. 2022). With the current speed of genomics, pharmacogenomics, and digital health technologies, it is difficult to keep up even for physicians who often lack the training necessary to guide patients on risk profiling and health monitoring (Rothstein 2017). Moreover, patients may have legitimate reasons for not complying with the imperative of preventive medicine to seek as much health information as possible. A study based on focus groups in the U.S. shows that the fraction of individuals *not* interested in the return of results from exome and whole genome sequencing was higher among self-identified African American participants, compared to non-African American participants, which the authors partly attribute to past experiences of injustices in research participation and clinical practice, as well as “pervasive and daily stress of being an African American” (Yu et al. 2013 p. 1072). Patients differ in their capacities to deal with, and preferences to know about, future diseases (Rothstein 2017), and estimates of expected benefits of PM have to account for the complexity of the wider social context of intended target populations (Vogt and Green 2020).

Despite good intentions to minimize health disparities through a focus on individualized prevention, the participatory approach of preventive PM implies an increasing responsibilization of individuals and potential stigmatization of health status. Not all patients have the resources to interpret risk information, navigate in digital healthcare spaces, or take the preventive actions that proponents of PM considered appropriate (Juengst et al. 2016). At the societal level, the emphasis on individual risk could thus destabilize the commitment to sharing risk and costs, regardless of what resources people have or how they decide to live their lives. We by no means deny that genetic risk profiling and (self)monitoring of health status is experienced as beneficial by some, as the popularity of online genetic testing and health apps indicates. But as long as convincing evidence for the effectiveness of individualized strategies in the prevention of”lifestyle diseases” is lacking (Vogt et al. 2018; 2019) and the challenges of structural inequality remain unaddressed, public health resources might be better spent on structural public health strategies (Valles 2018).

## Discussion: time to reconsider PM?

Already in 1971, the British doctor Julian Hart formulated what is now known as the *inverse care law* stating that “[t]he availability of good medical care tends to vary inversely with the need for it in the population served” (Hart 1971, p. 405). Hart emphasized that this risk is particularly high when medical care is exposed to market forces. Since PM is driven in part by commercial enterprises that seek for-profit data collection and use opportunities, and since some PM-applications target consumers outside the healthcare system, it is particularly important to consider what scenarios are likely to materialize. Structural factors are also particularly important to discuss in this context due to the high costs of targeted therapies and the emphasis on “patient participation”, which often shifts the responsibilities of making sense of health information and taking disease-preventive actions onto the individual. In the previous sections, we have emphasized how structural factors can influence the impact of PM in terms of reducing or aggravating structural inequality.

The key question in this discussion is who can be expected to benefit from PM. A recent article based on interviews with GPs in Germany associates PM with an increased risk of *medical Matthew effects* where healthcare resources further shift towards the “worried well” (Gabriels and Moerenhout 2018). A similar concern was highlighted also in an early report on genetic technologies by the Danish Council of Ethics:

“In a possible future where many people search for insight into their genetic dispositions to disease, the consequence can be a spate of overdiagnosis and overtreatment, and hence a drain on resources as a result of resources in the health services being deployed on uncertain risk states rather than more serious disorders. This would be tantamount to an unfair redistribution of the health services’ limited resources” (Danish Council of Ethics 2012, p. 66).

The Council here highlights that the harm can be twofold, as test results of uncertain benefits can lead to overmedicalization and overdiagnosis of healthy individuals (see also Vogt et al. 2019), while those in need of care are deprived of healthcare resources. Some Danish doctors report on increasing demands for follow-up testing and counseling from individuals, who have bought genetic tests online or collected data via self-monitoring technologies (Aagaard 2019; Videbæk et al. 2019). Confronted with uncertainty about the clinical utility of many genetic tests, as well as the lack of specialized training in genetics or resources for advising patients on these matters, the GPs call for regulation and oversight. Such calls highlight how PM, even when driven from the “demand side”, can aggravate or drive health disparities. This underscores the urgency of discussing how to benefit from PM without undermining existing healthcare structures.

It is a common concern that PM and its focus on individual characteristics challenge the principle of solidarity within public healthcare systems (e.g., Fleck 2022). Whether it does or does not, is in part a political decision. Existing universal healthcare systems were set up with the deliberate commitment to ignore differences in individual risk when determining people’s contributions to the system (Ter Meulen et al. 2013). The principle of solidarity means that people contribute as they can, and receive support as they need—regardless of their ability to pay, how they live their lives, and irrespective of the likely costs that they will incur for the system. The fact that PM enables the stratification of patients in much more granular ways than was previously possible does not change this. Just because we now know more about people’s individual risks does not change the principle that this risk does not determine people’s contributions. However, it changes the diversification of preventive, diagnostic, and therapeutic pathways for patients according to molecular and other characteristics, with the corresponding diversification of cost. As noted earlier in this article, some treatments have become so expensive that they pose the question of whether a system that seeks to give everyone what they need can be upheld at all.

Another notable change is the intensified focus on individual risk in preventive medicine. While testing for risk factors (e.g., blood cholesterol) in individuals has been part of preventive medicine for decades, PM greatly expands the efforts by including finer-grained measures and continuous monitoring at increasingly earlier stages (e.g., DanskeRegioner 2015b; Price et al. 2017). Although PM holds great potential for more accurate risk profiling, it also raises concerns about risks of “blaming the victims” of structural and systemic injustices by “unnecessarily imposing group harms like stigmatization and diverging public health resources from efforts to address the underlying social determinants of health disparities” (Juengst et al. 2016, p. 29). A related concern is how the focus on individualized risk can support “neoliberal efforts to relieve society of collective responsibilities for health care equity” (Ibid, p. 22), which underscores the importance of discussing how the technologies can be used to benefit the intended target groups. Based on our analysis, we make the following concluding remarks with suggestions for how to mitigate the risk of increasing structural inequality.

First, robust scientific development of PM requires higher diversity of data donors and clinical study participants. Here it should be highlighted that not all biases are bad: oversampling underserved populations can be mandated if the aim is to rectify existing biases (Pot et al. 2021). We, therefore, welcome initiatives to balance the lack of diversity in data to build risk scores and identify biomarkers. Yet, we caution against a simplified focus on diversity of genetic data as a means for medical inclusion as this risks reifying or naturalizing racial categories while neglecting socioeconomic and other demographic factors (see point five below). Research inclusivity thus requires considerations of multiple factors beyond genetic diversity that may influence the relative benefits of PM. Moreover, we emphasize that ensuring inclusivity is a complex problem that should be understood in the context of wider disparities and barriers for some subpopulations (Sabatello and Appelbaum 2017). Commenting on the implied reciprocity of the “gift” metaphor of data donation, ethicist Sandra Lee (2021) stresses the importance of addressing “social differences as context for gift giving” (p. 60). Efforts to improve data diversity should be complemented by an ethical commitment to reciprocity also in *benefit sharing*.

Second, since PM is fundamentally dependent on access to integrated sources of genomic and health data, trust relations between the public and health institutions must be restored and maintained. Also here, action needs to go beyond the symbolic, and also beyond initiatives to ensure informed consent and data safety. While these issues are crucial in avoiding harm at the individual level, we also need to attend to group harms, e.g., when health data are used to reinforce misperceptions about marginalized populations (Sabatello et al. 2022) or when sharing of health data stimulates market interests that are counter to the interests of data donors. We, therefore, believe that ensuring trust in data handling will require legislative changes to ensure that the public will benefit from public–private partnerships. At present, public healthcare systems make resources available for clinical trials that contribute to the development of precision treatments. Yet, these treatments are often so highly-priced that the same systems cannot afford them (Hillersdal and Svendsen 2022), or the trials may further stimulate global inequalities and differences between countries with powerful pharmaceutical industries. We also highlighted how the combination of increasing prices and high public expectations to PM put public healthcare systems under pressure and may stimulate a market for additional private health insurance. As this may lead to downstream increases in structural equality, even in highly inclusive systems, we find this issues an important one to address. To counter such effects, ethics approval of protocols for PM clinical trials should be conditioned on clear public returns for data donation and trial participation. This could be done by placing more emphasis on evaluating the cost-effectiveness of treatments and the willingness of pharmaceutical companies to comply with cost regulation and the ethical requirement of benefit sharing. A further suggestion is to implement oversight of secondary uses of health data from community-based efforts. Greater inclusion of communities in data oversight can improve the understanding of social as well as epistemic context of the data and data donors, thus minimizing the risk of unjust uses of health data.

Third, and following on the two points above, it is critical to work towards equal access to affordable high-quality healthcare and PM services. Without this structural basis for the intended aims of PM, the claim of making up for past wrongs is merely symbolic, and may even aggravate existing disparities. The previously cited *Nature* editorial (2021) emphasizes that greater diversity in genetic data diversity can be obtained by making participants “well informed and aware of the value that these studies may have for improving health in their communities” (p. 737). But such promises of health benefits are empty if underrepresented groups do not get access to the resulting genomic applications. Large-scale investments in PM should not overlook the fact that, in many contexts, access to basic healthcare cannot be taken for granted, and that experienced barriers to healthcare access influence expectations to research benefits (Kraft et al. 2018). Moreover, since PM creates a market for particularly costly treatments and additional services, PM may primarily benefit those who already have the best opportunities and stimulate competing private markets that are of disproportionate benefit to the public. Even in countries where universal healthcare coverage exists, it is important to consider initiatives to avoid the destabilization of egalitarian health policies, as discussed below.

Fourth, our analysis shows that PM can challenge a fair and equitable prioritization of healthcare resources. What is “fair” depends not only on distributive justice but also on the business model of public–private partnerships and the pricing of drugs. The high price of targeted treatments, such as Zolgensma, calls for a critical assessment of the cost-efficiency of PM treatments and procedures for price regulation. Similarly, as uncertainties about the effects of individualized prevention remain, there is a need for evidence-based guidelines on how to evaluate “precision screening” programmes that are often targeted directly at consumers (Vogt et al. 2019). The updated Danish strategy for PM flags the need to consider the relative cost-efficiency by listing, as one of six principles, the condition that PM treatments offered should be *socio-economically sustainable* (Sundhedsministeriet og Danske Regioner 2021: 12). We welcome the increased attention to the problem of opportunity cost, but find it concerning that policy reports often state the expected benefits of PM without any evidence to justify the promises made. Sound prioritization of healthcare resources requires more transparency about the expected costs and benefits for different stakeholders (including patients), as well as information about how these are estimated.

Fifth, the realization of PM hinges on the possibility of managing the challenge of collecting and making sense of unprecedented amounts of data. Addressing this challenge requires greater acknowledgment of the resources needed for data work, educational training of health professionals, and additional support and counseling for citizens who cannot all be expected to have high levels of (digital) health literacy. Hence, expected benefits should not be extrapolated from the enthusiasm of patients who are "uncommonly tech savvy, health literate, self-directed, information seeking, English fluent, health focused, and well insured” (Rothstein 2017, pp. 277–278). Moreover, as debates about “the right not to know” illustrate, patients may have different preferences for how much health information they would like to receive. Patients may also have legitimate reasons to not comply with the norms of preventive medicine, e.g., if they have other priorities or face challenges in life. This underscores the importance of accounting for the complex social context of the intended target populations, leaving open whether PM solutions are the ones that best address the most pressing problems (Vogt & Green 2020).

Finally, we need to systematically consider the structural causes of health inequities. To significantly improve public health, a focus on individual (genetic) differences will not suffice (Olstad and McIntyre 2019). Health is determined by multiple factors over which individual people have little or only marginal control. An important next step will be to include a broader spectrum of causes and intervention strategies in predictive models in PM. An approach that considers the health effects of other measures, such as housing, finance, and other policy fields, would help to bolster a more equitable foundation for PM (Ollila 2011). This requires data collection strategies that to a higher extent include proxies for structural determinants of health disparities, such as lack of access to health care, inadequate housing, and environmental factors (Khoury et al. 2022). Taking this one step further, social scientists Senier, Brown, Shostak, and Hanna have suggested the term *socio-exposome* to address environmental injustice by including also “the sociopolitical conditions and inequalities that allow hazards to continue unchecked” (Senier et al. 2017 p. 107; see also Shostak 2004; 2013). The authors further emphasize that closer collaboration between biomedical researchers and social scientists could help mitigate the risk of molecularizing complex social phenomena, understood as the risk of “reducing the social experiences that condition population-level variation in exposures to individual-level molecular-level differences” (Ibid p. 107). In addition, we note that bioethicists, social scientists, and philosophers of science can contribute with a critical examination of the benefits and challenges of proposed applications, such as individualized disease prevention (e.g. Vogt et al. 2019). We contend that individualized strategies should not be prioritized in contexts, where convincing evidence is lacking for their cost-effectiveness, and where structural interventions can be expected to lead to similar, or better, results, without involving stigmatization and responsibilization of individuals (Juengst et al. 2016).

## Concluding remarks

PM entails a potential to improve the effectiveness of medical treatments and constructively points to the harms caused by standard treatments that fail to account for individualized factors. But whereas the vision for PM is about prioritizing what is best for individual patients, how PM can be expected to benefit a very small subset of patients is often neglected. The consideration of the effects of personalization for different groups and in different countries exposes how medical strategies must always be evaluated in relation to the healthcare systems in which they are implemented, and in the context of wider social and economic factors.

In this paper, we discussed how the realization of PM may play out differently depending on the structure of existing healthcare systems. We argued that the problem of structural injustice cannot be solved solely by increasing the precision of medical technologies, or by enhancing the inclusiveness of PM research. Addressing this problem depends on the willingness to address existing structural barriers that hinder progress and factors that may challenge fairness in the distribution of healthcare resources. The examined issues intersect with the broader question of the epistemic and ethical implications of the increasing focus on individual differences as a focal point for medical development.

For PM to be implemented in a way that does not increase existing inequities, it is necessary to address existing health disparities and gaps in healthcare systems. The organization of healthcare systems can either exacerbate or mitigate potential negative effects, in particular the personalisation of risk and increased inequality in terms of cost and access. While we do not see a health-for-all model, as seen in Austria or Denmark, as a solution to all problems, many foreseeable problems can be avoided through systems that ensure a solidaristic sharing of cost and risk. Supporting and maintaining such systems is particularly important in PM, where “medical Matthew effects” are driven not only by for-profit market mechanisms but also by unrealistic expectations of genetic technologies. We offered six suggestions for how to mitigate the risk of increasing structural inequality, which call for a realistic assessment concerning expected returns as well as attention to factors that influence who will benefit from PM in the future.

## Footnotes

1 As an anonymous reviewer helpfully pointed out, the impact of ethnicity on genetic test results can differ. For example, it is often relevant to distinguish between germline (hereditary) pathogenic variants and acquired somatic mutations in a tumor, although recent studies indicate that also the rate of somatic mutations can depend on ethnicity, among other factors (e.g., García-Nieto et al. 2019; Holowatyj et al. 2023). Our concerns about inclusivity are wider, since treatment effects cannot be uncritically extrapolated across populations, e.g., if the groups differ in rates of co-morbidity or options for access to healthcare services and sick leave. For this reason, our main aim is to address the implications of wider structural factors in this paper.

2 See National Center for Health Statistics: https://www.cdc.gov/nchs/fastats/electronic-medical-records.htm, accessed July 14, 2022.

3 Yet, with all its strengths, the Austrian healthcare system is not always well equipped to address the needs of “a-typical users”. For example, the healthcare system has not adjusted to accommodate the different health needs of newcomers who have suffered traumatising experiences of conflict and violence. Structural and implicit biases disadvantage people who do not speak German, or those who may be hesitant to articulate their needs to healthcare providers due to cultural, religious, or other personal reasons (Spahl and Prainsack 2021).

4 For more information, see https://trialnation.dk , as well as (Ministry of Foreign Affairs 2014; Sundhedsministeriet og Danske Regioner 2021)
