# Correction to: Precision medicine and the problem of structural injustice

**DOI:** 10.1007/s11019-023-10173-9

**Published:** 2023-08-28

**Authors:** Sara Green, Barbara Prainsack, Maya Sabatello

**Affiliations:** 1 Section for History and Philosophy of Science, Department of Science Education, University of Copenhagen, Niels Bohr Building (NBB), Universitetsparken 5, Copenhagen Ø, 2100 Denmark; 2 Centre for Medical Science and Technology Studies, Department of Public Health, University of Copenhagen, Oester Farimagsgade 5, Copengagen, 1014 Denmark; 3 Department of Political Science, University of Vienna, Universitätsstraße 7, Vienna, 1010 Austria; 4 School of Social and Political Sciences, Faculty of Arts and Social Sciences, University of Sydney, Camperdown, NSW 2006 Australia; 5 Center for Precision Medicine and Genomics, Department of Medicine, Columbia University, New York, USA; 6 Division of Ethics, Department of Medical Humanities and Ethics, Columbia University, New York, USA

**Medicine, Health Care and Philosophy (2023) 26:433–450**

10.1007/s11019-023-10158-8

In the original publication of the article, the price of the treatment called Zolgensma in the fourth paragraph under the heading “Prioritization of healthcare resources” was published incorrectly. The sentence should read as “1.9 million US$”.

The original article has been corrected.

